# Accurate Analysis of Tumor Margins Using a Fluorescent pH Low Insertion Peptide (pHLIP)

**DOI:** 10.3390/ijms10083478

**Published:** 2009-08-04

**Authors:** James Segala, Donald M. Engelman, Yana K. Reshetnyak, Oleg A. Andreev

**Affiliations:** 1 Physics Department, University of Rhode Island, 2 Lippitt Rd., Kingston, RI 02881, USA; E-Mail: segalaj@egr.uri.edu (J.S.); 2 Department of Molecular Biophysics and Biochemistry, Yale University, P.O. Box 208114, New Haven, CT 06520, USA; E-Mail: donald.engelman@yale.edu (D.M.E.)

**Keywords:** tumor border, fluorescence-guided surgery, pHLIP, tumor targeting, computational algorithm

## Abstract

The recurrence of certain cancers remains quite high due to either incomplete surgical removal of the primary tumor or the presence of small metastases that are invisible to the surgeon. Near infrared (NIR) fluorescence imaging might improve surgical outcomes by providing sensitive, specific, and real-time visualization of normal and diseased tissues if agents can be found that discriminate between normal and diseased tissue and define tumor margins. We have developed a new approach for revealing tumor borders by using NIR fluorescently labeled pH Low Insertion Peptide (pHLIP) and have created a computational program for the quantitative assessment of tumor boundaries. The approach is tested *in vivo* by co-localization of GFP-tumors and NIR emission from the fluorescently labeled pHLIP, and it is found that boundaries are accurately reported and that sub-millimeter masses can be detected.

## 1. Introduction

In contemporary medicine, image-guided surgery has been introduced to maximize tumor excision and minimize collateral damage. Surgeons usually rely on MRI images obtained before surgery to aid in the removal of a tumor, and recently introduced fluorescence-guided procedures are considered to be very promising [1–6]. Most tumors do not have precise boundaries, and small, distinct metastases can exist near the main tumor mass, so labeling might improve outcomes, particularly if small masses can be revealed. But, there is a significant limitation in the application of optical imaging for whole-body scans due to poor light penetration through tissues. Optical techniques might be useful if the fluorescence signal is collected from a surface, as in a surgical setting where diseased tissue is exposed to view, but only if an optical imaging agent can preferentially and accurately target cancer cells.

Traditionally, receptors and enzymes overexpressed in cancer cells have received primary consideration as appropriate biomarkers for targeted imaging and/or therapy [7–9]. Unfortunately, the variability of the cells in human cancers may limit approaches based on targeting specific cancer cell receptors [10,11]. Recent studies of gene expression in cancer cells indicate that a number of genes are up- or down-regulated in individual cells within a tumor, such that cell surfaces in a tumor are heterogeneous [12]. It is therefore problematic to rely on any single tumor biomarker even for one type of cancer, and a need exists to seek other strategic options. Tumor acidity, which is a feature of most solid tumors, might be an attractive cancer biomarker if means can be found to specifically target cells in low pH environments [13].

We have demonstrated that pH Low Insertion Peptide (pHLIP), targets acidic tissues *in vivo*, and can be used to localize molecules at cell surfaces or to translocate otherwise cell-impermeable cargo molecules into cells [14–17]. pHLIP, when conjugated with various fluorescent and positron emission tomography imaging agents, can accumulate in tumors of various types established in mice [14–16]. The accumulation of pHLIP in tumors depends directly on the extracellular acidity of the tumor environment regardless of origin and can be regulated by pH. Targeting employs direct insertion of monomers across cell membrane lipid bilayers and should not be subject to the same heterogeneity influences as marker targeting, since the mechanism does not involve the participation of membrane proteins or endocytotic uptake. Here we demonstrate that fluorescent pHLIP targets cancer cells to effectively mark their boundaries, and present a specially designed computational program that reveals tumor borders with high accuracy.

## 2. Results and Discussion

### 2.1. In vivo fluorescence imaging

**Figure 1.**
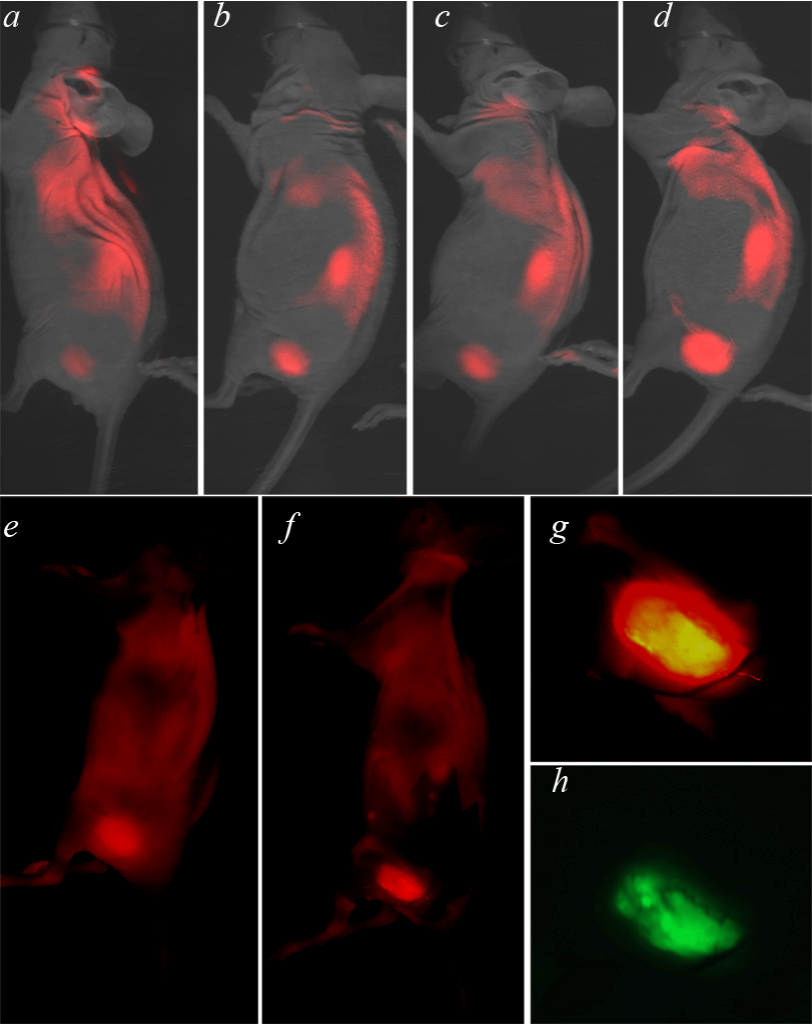
Overlay of whole-body NIR fluorescence and light (photo) images of a mouse bearing a tumor in its right flank is presented. The tumor was established by the subcutaneous injection of GFP-HeLa cells. Cy5.5-pHLIP was given as a single tail vein injection. Imaging was performed 4 (*a*), 24 (*b*), 48 (*c*) and 72 hours (*d*) after injection of Cy5.5-pHLIP. Whole-body NIR fluorescence images of mouse bearing GFP tumor 72 hours after Cy5.5-pHLIP injection (*e*), and the same mouse with partially removed skin (*f*) are shown. Magnification of the tumor site: NIR fluorescence of Cy5.5-pHLIP (*g*) and GFP-tumor (*h*).

Our goals are to test the ability of fluorescent pHLIP to reveal tumor borders and to develop a computer program for the quantitative assessment of the co-localization of GFP-tumors and the pHLIP signal *in vivo*. We used HeLa cancer cells expressing green fluorescent protein (GFP), which allowed clear visualization of the tumor and its border. The tumor was established by subcutaneous injection of GFP-HeLa cells in nude mice. When the tumor reached 3–5 mm in diameter, fluorescent pHLIP was given as a single i.v. injection in the tail vein. We used Cy5.5 covalently conjugated to the *N*-terminus of pHLIP, which remains outside a cell when the peptide inserts to form a transmembrane helix at low pH. Whole-body fluorescence imaging was performed at 4, 24, 48 and 72 hours after Cy5.5-pHLIP injection. The tumor data are shown in the specific tumor targeting image (Figure 1, both the tumor, and the kidney are seen in the images). The fluorescence signal decreases much more slowly in tumors than in normal tissue, resulting in an increase of contrast index (CI) with time (CI=6–7,72 hours after Cy5.5-pHLIP injection). pHLIP stains the skin somewhat, since the pH of skin is lower than that of most normal tissue (7.4). Therefore, a fluorescence (and scattered light) background is created by the skin. The image becomes much clearer if the skin is removed (compare Figure 1e and 1f, where the skin was partially removed). Figure 1g and 1h show magnified views of NIR and GFP images of a tumor site. The GFP expressed in the tumor cells allows clear visualization of the tumor and its border. To get precise information about tumor margins from the NIR fluorescence image we have created a special computational algorithm.

### 2.2. Computational algorithm for the identification of tumor borders: EdgeFinder

The EdgeFinder algorithm was developed to examine *in vivo* co-localization of GFP and NIR CY signals, but may have continuing applications. The algorithm consists of the following sub-sections: application of edge detection filters, image background removal, and boundary detection. Before detecting the boundary of tumor, the images are processed using two different filters for the detection of edges. First, a Gaussian filter (Equation 1) is applied to smooth transitions and remove speckles from the image:

(1) $$G(x,y)=\frac{1}{2\pi \sigma^{2}}e^{\frac{x^{2}+y^{2}}{2\sigma^{2}}}$$

Next, a gradient filter (Equation 2) is used to accentuate the areas of large image intensity variations, which correspond to tumor edges:

(2) $$\Delta I(x,y)=\frac{\partial I(x,y)}{\partial x}\hat{i}+\frac{\partial I(x,y)}{\partial y}\hat{j}$$

Combination of the (Equation 2) and (Equation 1) gives the filter kernel:

(3) $$\Delta G(x,y)=\frac{\sqrt{x^{2}+y^{2}}}{2\pi \sigma^{4}}e^{\frac{x^{2}+y^{2}}{2\sigma^{2}}}$$

**Figure 2.**
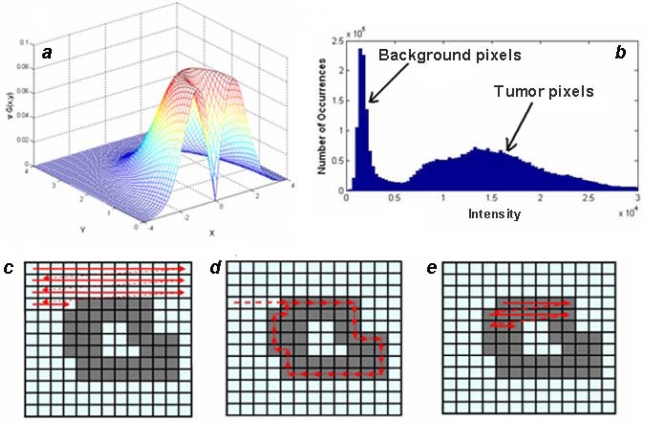
*a*) An example of the gradient of a Gaussian filter with a standard deviation of 1 and window size of 4. *b*) Histogram of intensities for an image containing multiple tumor sites. The background intensity is determined as the intensity bin with the largest number of pixels. *c–e*) The algorithm for the detection of boundaries on a binary image is shown. Starting from the home position it scans until an ‘on’ pixel is found (*c*), then walks the perimeter of the boundary keeping the ‘off’ pixels to the left (*d*), then steps *c* and *d* are applied to the entire image to detect all sub-boundaries (*e*).

Equation 3 is applied to the entire image as a convolution kernel with a specified window size. The standard deviation σ, and the convolution window are varied to obtain the best results. Figure 2a demonstrates a typical filter configuration.

Usually, NIR and MRI images contain low level background noise that should be removed from an image before edge detection can take place. The intensity of the noise is determined using a histogram of the pixel intensities in the image. It is computed by counting the number of pixels with intensities that fall within evenly spaced bins as illustrated in Figure 2b. The intensities corresponding to the bins with the largest counts are considered to be the background level, which is subtracted from the image.

The next step is boundary detection. Images filtered using gradient functions have high intensity strips corresponding to boundaries. Detection of these boundaries is done on a binary image where a pixel is either on or off. After filtering and background subtraction, the image is converted to binary by comparing each pixel intensity with a threshold value, chosen using Otsu’s Method [18], and making the pixel either ‘on’ if the intensity is greater than the threshold, or ‘off’ if the intensity is below the threshold. The algorithm generates a list of boundaries for a binary image, as illustrated in Figure 2c–e, and proceeds by scanning the image until an ‘on’ bit is detected, which is considered the first pixel in a boundary. Keeping ‘off’ pixels to the left, the boundary is walked until the first pixel is returned to. Scanning continues until all areas of the image are processed including the area within a boundary.

### 2.3. In vivo co-localization of NIR and GFP fluorescence

**Figure 3.**
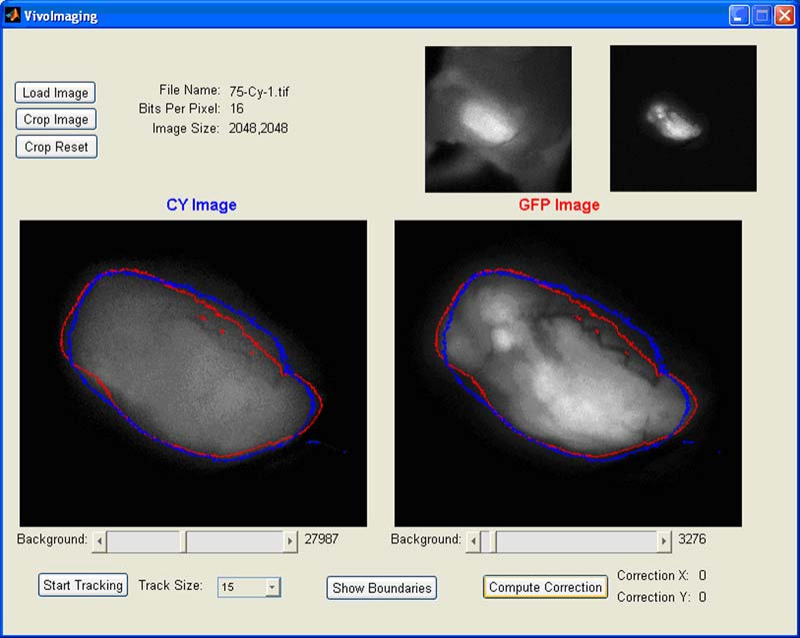
The output window of the EdgeFinder program is presented. It contains the original GFP and NIR fluorescence (CY) images, and processed images with computed tumor borders. Blue and red colors are used to show tumor borders calculated from the NIR and GFP fluorescence images, respectively.

**Figure 4.**
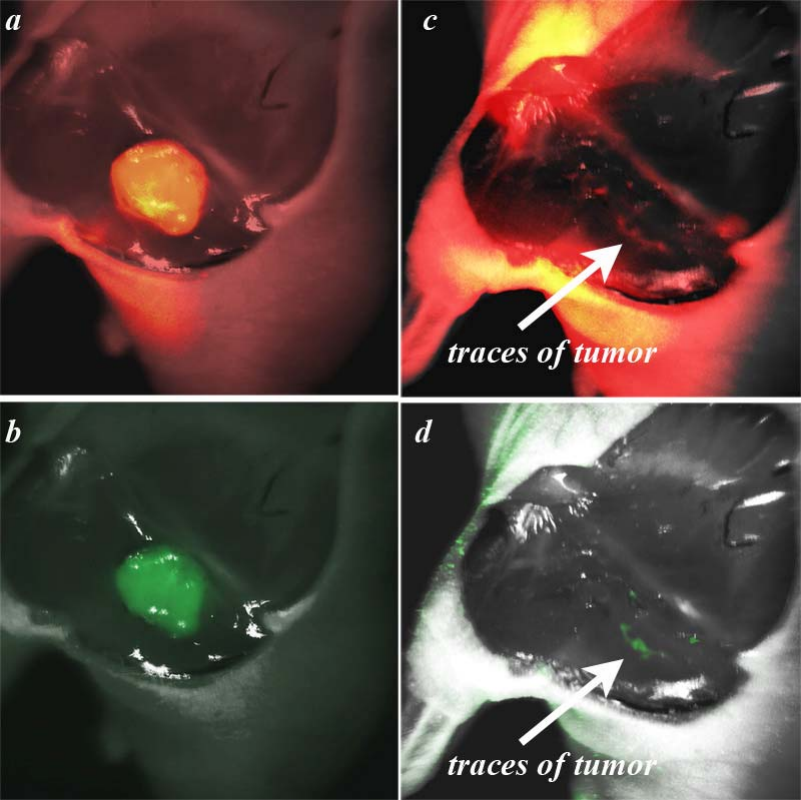
Overlay of photo and fluorescence images of tumor site before (*a–b*) and after (*c–d*) tumor removal. NIR Cy5.5-pHLIP emission with partial skin removal is shown in *a* and *c*; GFP tumors are presented in *b* and *d*.

We applied the EdgeFinder algorithm for the analysis of the GFP and NIR Cy5.5 fluorescent images presented in Figure 1g and 1h. The program output window is presented in Figure 3, where the tumor borders calculated from the NIR Cy5.5-pHLIP and GFP-fluorescence images are shown as blue and red lines, respectively. Tumor borders established from the GFP and NIR images are in excellent agreement with each other. We explored how well the tumor was removed during surgery. The GFP and NIR fluorescence of a tumor are overlaid with a photo of the tumor site and presented in Figure 4a and b. We performed “surgery” and removed the main mass of the tumor, but some traces, invisible to the naked eye, could be visualized by GFP fluorescence (Figure 4d). The same spots were seen using the pHLIP labeling with NIR settings (Figure 4c).

**Figure 5.**
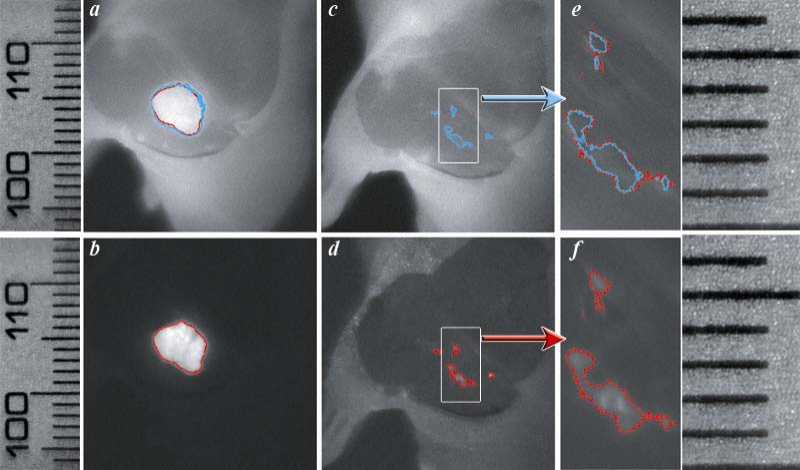
Tumor border calculated from the fluorescence images of a tumor site before (*a–b*) and after (*c–d*) tumor removal. NIR Cy5.5-pHLIP emission images with skin partially removed are shown in *a*, *c* and *e*; GFP tumors are presented in *b, d* and *f*. The magnified area (3.3 times) of tumor removal is presented in *e* and *f*. Cyan and red colors are used to show the tumor border calculated from the NIR and GFP fluorescence images, respectively (ruler is in mm).

The EdgeFinder algorithm was applied to show the tumor border before and after surgery (Figure 5).

Tumor margins obtained by the analysis of GFP and Cy fluorescence pictures are in close agreement with each other. Note that the fluorescent pHLIP is capable of detecting even sub-millimeter pieces of tumor remaining after removal of the main mass (see Figures 5e and f and the corresponding ruler in the right part of Figure 5).

The data show that fluorescently labeled pHLIP might be useful for fluorescence-guided surgery. The use of pH-sensitive fluorescent dyes that give a higher signal at low pH than at normal pH might enhance the tumor/background ratio and could be even more beneficial in applications to fluorescence-guided surgery. The EdgeFinder algorithm could be useful for defining tumor margins from NIR fluorescence images. We have demonstrated that tumor borders established from the Cy5.5 fluorescence images are in close agreement with the borders found by analysis of the constitutive GFP fluorescence of the cell in the tumor.

## 3. Experimental Section

The pHLIP peptide (ACEQNPIYWARYADWLFTTPLLLLDLALLVDADEGT) was prepared by the solid-phase synthesis at the W.M. Keck Foundation Biotechnology Resource Laboratory at Yale University. Cy5.5 mono maleimide (GE Healthcare) was conjugated with the SH-group of Cys at the N-terminus of pHLIP by incubation in DMSO in ratio of dye:peptide of 2:1. The conjugated peptide was purified from free dye and unreacted peptide by HPLC. The purity of the product was accessed by HPLC and SELDI-TOF masspec. The concentration of the labeled peptides was determined by absorption, ε_280_=13,940 M^−1^ cm^−1^ for pHLIP and ε_673_=190,000 M^−1^ cm^−1^ for Cy5.5.

Tumors were established by subcutaneous injection of adult athymic nude mice with HeLa cancer cells expressing green fluorescence protein (2×10^7^ cells/flank/0.2 mL). Three weeks after injection, each tumor typically reached 3–5 mm in diameter. Cy5.5 pHLIP was given as a single i.v. tail vein injection (0.7 mg/kg). Whole-body fluorescence imaging 4, 24, 48 and 72 hours post injection was performed on an FX Kodak *in-vivo* image station. During the imaging procedure, animals were under gas anesthesia. All animal studies are conducted in accordance with the principles and procedures outlined in the National Institutes of Health Guide for the Care and Use of Animals.

The program for tumor border calculation has been created in MatLab and the computational program can be freely obtained from the authors (the University of Rhode Island has copyrights for the program).

## 4. Conclusions

Fluorescently labeled pHLIP can target tumors and discriminate between normal and diseased tissue. A computational algorithm, “EdgeFinder”, for analyzing the distribution of fluorescent signals in tissue allows one to detect tumor margins accurately. Submillimeter masses can be accurately found and mapped. This imaging approach might improve human cancer surgery by providing sensitive, specific, and real-time visualization to enable conservative and effective removal of diseased tissues.
